# GHB: a life-threatening drug complications and outcome of GHB detoxification treatment—an observational clinical study

**DOI:** 10.1186/s13722-023-00414-w

**Published:** 2023-10-21

**Authors:** Peter Neu, Heidi Danker-Hopfe, Robert Fisher, Felicitas Ehlen

**Affiliations:** 1 Jüdisches Krankenhaus Berlin—Clinic for Psychiatry and Psychotherapy, Heinz-Galinski-Str. 1, 13347 Berlin, Germany; 2 Competence Center for Sleep Medicine, Charité—Universitätsmedizin Berlin, Campus Benjamin Franklin, Hindenburgdamm 30, 12203 Berlin, Germany; 3 Stepney and Wapping CMHT, Queen Mary University, Wolfson Institute for Preventive Medicine, 68 Glasshouse Fields, London, E1W 3AB UK; 4 Charité—Universitätsmedizin Berlin, Clinic for Psychiatry and Psychotherapy, Bonhoefferweg 3, 10117 Berlin, Germany

**Keywords:** GHB, Detoxification, Delirium, Intensive care unit

## Abstract

**Background:**

GHB (gammahydroxybutyrate) and its precursors are popular recreational drugs due to their sedative, anxiolytic and sexually stimulating effects. Their use has been steadily increasing in recent years. The detoxification process is complex and prone to high rates of complications while little is known about the pathophysiology. This study aims to elucidate the characteristics of GHB-addicted patients and to evaluate the risks and complications of GHB withdrawal treatment.

**Methods:**

This observational study describes prospectively the socioeconomic status, clinical history and course of inpatient detoxification treatment of a group of 39 patients suffering from GHB substance use disorder. Detoxification treatment took place in a highly specialized psychiatric inpatient unit for substance use disorders.

**Results:**

GHB patients were characterised by being young, well-educated and by living alone. More than 50% of the patients had no regular income. The patients were male and female in equal numbers. Detoxification treatment was complicated, with high rates of delirium (30.8%) and high need for intensive care (20.5%).

**Conclusions:**

In our sample, GHB users were young, well-educated people and male and female in equal number. Detoxification proved to be dangerous for GHB-addicted patients. The presence of delirium and the need for transfer to an intensive care unit during detoxification treatment was extraordinarily high, even with appropriate clinical treatment. The reasons for this remain unknown. Therefore an intensive care unit should be available for GHB detoxification treatment. Further studies are needed to evaluate the options for prophylactic treatment of delirium during detoxification.

## Background

GHB (gammahydroxybutyrate) and its precursors GBL (gamma-Butyrolactone) and 1,4 BD (1,4-Butandiole) have gained popularity as recreational drugs. Due to their sedative, anxiolytic and sexually stimulating effects [1]. The lifetime prevalence of consumption used to be fairly low (0.1–13% worldwide) [1, 2], however, recreational use of GHB is rising, especially in the clubbing scene [3]. The European Drug Emergencies Network (Euro-DEN) [4] is a European Commission funded project which has set up a network of sentinel centres with toxicological expertise across Europe to collect systematic data on presentations to emergency rooms (ER) with acute drug and psychoactive substances toxicity. The Euro-DEN network consists of 16 sentinel centres (Barcelona, Basel, Copenhagen, Drogheda, Dublin, Gdansk, London (two centres), Mallorca, Munich, Oslo (two centres), Paris, Parnu, Tallinn and York) in 10 European countries (Denmark, Estonia, France, Germany, Ireland, Norway, Poland, Spain, Switzerland and the UK). Dines and colleagues presented the main Euro-DEN findings from 12 months of data collection (October 2013 through September 2014) from these sentinel centres. GHB/GBL were the fourth most common drug overall. Notably over 90% of presentations associated with GHB/GBL were from the London, Oslo and Barcelona centres. Two centres had no presentations at all involving GHB/GBL. Patients self-presented to ER due to suspected intoxication and not due to withdrawal symptoms. The drugs were recorded based on the patient’s self-reporting and/or the physician’s interpretation of the patient’s clinical features. Nevertheless, GHB seems to play an increasing role in the drug scene, but data of GHB-addicted patients seeking detoxification and relapse prevention treatment are lacking.

GHB is a substance found naturally in the human central nervous system and in organs such as liver, kidney, heart, bones and brown fat. In humans the highest concentrations are found in the basal ganglia with binding sites in the same location, and in the cortex, hippocampus, mid-brain and substantia nigra [5]. GHB is both a prodrug and metabolite of the neurotransmitter GABA (gamma-aminobutyric acid) [6]. In the brain GHB is synthesized from GABA in cells containing glutamic acid decarboxylase, the marker of GABAergic neurons. These receptors are thought to regulate GABAergic activities via a subtle balance between sensitized/desensitized states. Activation of GABA-A and GABA-B receptors induced by GHB’s conversion to GABA might be responsible for GHB’s anxiolytic and sedative effects [7]. GHB can also be synthesized in vivo from its prodrugs or precursors GBL and 1,4-BD, with GHB being the compound exerting the pharmacological effect [8]. Following exogenous (oral) administration, GHB is absorbed rapidly from the gastro-intestinal tract. The effects start about 15 min after an oral dose. The half-life time of GHB is in the range of 20 to 45 min [9]. This might explain why users report taking small doses of GHB every 2 to 3 h throughout the day. The conversion of GHB to succinic semialdehyde and entry into the Kreb’s cycle are the primary metabolic pathway for GHB Only 2–5% of GHB is eliminated unchanged in urine [10, 11].

Clinical effects become evident as early as 5 min after ingestion, reaching their peak after 30–60 min. GHB can induce a dose-dependent stimulant or sedative effect, adding to its recreational appeal [12]. This dual effect is associated with euphoria, disinhibition, sensory and sexual arousal at lower doses, evolving into anxiolytic, narcotic and sedative effects and altered states of consciousness at higher doses. The dose–response window of GHB is narrow, and small dose increments can easily lead to life threatening overdoses [13].

Regular GHB use can lead to dependency within weeks. GHB withdrawal is known to cause autonomic dysfunction with severe CNS symptoms [10]. Abrupt withdrawal can lead to a range of neurological symptoms: tremor, anxiety attacks, confusion, seizures, and memory loss have all been described. Initial symptoms may progress to severe delirium with auditory and visual hallucinations and cardiovascular effects including tachycardia and hypertension. The withdrawal syndrome of GHB, GBL or BD closely resembles that of alcohol [14]. GHB detoxification can be very dangerous, entailing delirium, agitation and other neuropsychiatric features. These symptoms are more common and worse in patients with GHB withdrawal and withdrawal from its analogues compared to ethanol withdrawal. There is no established management strategy for patients presenting with acute withdrawal related to GHB dependence [15].

Most treatment regimens recommend use of benzodiazepines in the management of acute GHB withdrawal [16], but pharmaceutical GHB, baclofen [17], propofol [18], and barbiturates [19] have also been suggested.

In the work of Raposo Pereira and colleagues [12], people taking GHB were described as young, well-educated, living alone and predominantly male. However, GHB-users’ characteristics are still largely unknown. Knowledge of these characteristics could help to identify typical GHB-patients and enable healthcare institutions to develop special programs of treatment and prophylaxis.

Furthermore, in order to improve specific withdrawal programs it seems of importance to investigate both withdrawal complications and drop-out rates.

This study therefore aims to shed light on the socioeconomic and clinical history of GHB-addicted patients and the clinical course and complications of the detoxification process.

Our study aims to address the following issues:

- What are some socioeconomic characteristics and medical history of the individuals of our study group addicted to GHB?
- What is the risk of complications such as delirium and seizures during withdrawal from GHB?

## Methods

### Setting and treatment procedure

This prospective observational study was conducted in a highly specialized psychiatric inpatient unit. The unit is registered for qualified detoxification treatment of substance use disorders within Jüdisches Krankenhaus Berlin. The team consists of medical doctors, psychologists, specialized nurses, occupational therapists, physiotherapists and social workers. The qualified detoxification treatment (QDT) combines physiological treatment with psychotherapy, psychoeducation and relapse prevention. It is a three-step process. During detoxification the patients physically withdraw from the drug and, when needed, withdrawal symptoms are treated pharmacologically. In a second step, the patients attend a minimum of five psychotherapeutic group sessions and two psychoeducational group sessions. The third step prepares the patients for the transition to long-term follow-up treatment after discharge and includes attendance of five self-help groups in an outpatient setting. The average duration of treatment overall is between 12 and 16 days but may be significantly longer if withdrawal symptoms persist, as is often the case with GHB detoxification, or if the patient is in poor physical health. Patients seeking withdrawal treatment are screened in our outpatient department prior to admission. A physical examination is performed and the medical history is taken. The patients give consent to the treatment procedures in hospital.

The study was approved by the local ethical committee of Charité—Universitätsmedizin Berlin (reference number: EA1/235/21).

### Patients

Between March 2019 and September 2020 all patients who were admitted for a GHB-dependency were screened for study participation by the medical doctors responsible for the treatment and asked for participation. No incentives were offered. Data was collected prospectively by the study doctors (PN and FE). Due to the coronavirus pandemic the study was interrupted between March and June 2020 and resumed on 1st July 2020.

### Inclusion and exclusion criteria

Inclusion criteria were (1) Fulfilling criteria for GHB substance use disorder, (2) Patients agreed to an elective admission for detoxification treatment, (3) They had sufficient proficiency in the German language and (4) They had capacity to give informed consent. Exclusion criteria were (1) A diagnosis of substance use disorder of any sedative other than GHB, (2) Lack of capacity and insufficient command of German language. All patients gave written informed consent.

### Diagnostic criteria

We used DSM-V criteria [20] for diagnosis of substance use disorder and withdrawal criteria focusing on delirium, seizures and concomitant illnesses evaluated by an experienced psychiatrist.

### Treatment and participating staff

All patients received treatment as usual (see treatment procedure and medication). The attending doctors or health professionals were not blinded with regards to the study.

### Medication

All patients received medication as usual if eligible. Our GHB detoxification regime uses diazepam with an initial dose of 10–20 mg every 2 h according to physiological response. Assuming that the patients would take his or her last GHB right before entering the clinic, we started applying diazepam right after admission in order to reduce the risk of heavy withdrawal symptoms and delirium. Reduction started step by step under control of heart rate, blood pressure and symptoms of delirium.

For treatment of delirium we used benzodiazepines in combination with antipsychotics.

### Definition of complications

Diagnoses of complications like delirium and seizures were made by an experienced psychiatrist. There were no standardized criteria for referral to intensive care. In GHB-patients referral to ICU was usually indicated if the dosage of diazepam was above 140 mg in 24 h. The decision was made by the responsible consultant psychiatrist and physician on-call.

### Definition of successful treatment

Treatment was considered a success if a patient completed the program and remained abstinent until discharge. A break of the regular program by an admission to intensive care would be resumed after return to the regular ward.

The treatment was considered aborted if a patient was discharged against medical advice or was using substances and refusing to participate in the treatment program leading to a premature discharge.

### Data analysis

To address the questions as described in the introduction, a descriptive analysis was carried out. Categorical variables were summarized by frequency and percentage tabulation regarding the following clusters:

1. Socioeconomic history (age, sex, status of graduation, occupation and employment, partnership, children, history of imprisonment). Status of school graduation was categorised in four groups: attending school for 9, 10 or 13 years, the latter giving you access to all university degrees in Germany. Students attending school for 8 years or less leave school without any qualifications. Post-school education was categorised in four groups with rising status: none, undertaking apprenticeship or currently in university education, apprenticeship successfully finished, university degree completed. Employment was categorised in six groups (see Table 1).

**Table 1:** Socioeconomic data of cases

	GHB-patients N = 39
Age (mean/SD)	29.56/7.40
Female sex in %	43.6%
Graduation in %	
No graduation	2.6
9 years of school	25.6
10 years of school	25.6
13 years of school	46.2
Further education in %	
None	20.5
Apprenticeship/academical studies ongoing	20.5
Apprenticeship successfully finished	41.0
Academic studies successfully finished	18.0
Partnership (yes) in %	18.0
One or more children (yes) in %	7.7
Employment in %	
Financial support by parents	7.7
Unemployed	18.0
Welfare	25.6
Unskilled work	15.4
Employed	33.3
Pensioned	0
History of imprisonment (yes) in %	2.6

*SD* standard deviation

2. Clinical history (additional addictive diseases, additional psychiatric or non-psychiatric diagnoses, first age at onset of any addictive disease, occurrence of previous detoxification treatments, occurrence of previous dropouts, previous withdrawal seizures, occurrence of previous delirium, previous referral to ICU during QDT story of use of psychotropic substances and psychiatric diseases, age of onset of dependency, number of previous detoxifications and history of complications during previous detoxification treatments) (see Table 2).

**Table 2:** Clinical history

	GHB-patients N = 39
Diagnosis of second addictive disorder (active)	33.3%
Diagnosis of second addictive disorder (abstinent)	28.2%
Diagnosis of third addictive disorder (active)	2.5%
Diagnosis of third addictive disorder (abstinent)	15.4%
Additional diagnosis of any psychiatric disorder	20.5%
Additional non-psychiatric disorder	38.5%
First age at onset of any addictive disease mean/SD	25.33/7.58
One or more previous detoxification treatments	43.6%
History of previous dropouts	17.4
History of previous withdrawal seizure	5.0%
History of previous withdrawal delirium (yes)	23.1%
History of previous referral to intensive care during QDT (yes in %)	13.2%

*SD* standard deviation, *QDT* qualified detoxification treatment

3. Clinical course (occurrence of delirium, withdrawal seizures, need for intensive care and artificial ventilation, premature drop-out of the treatment program and discharge against medical advice) as indicated in Table 3.

**Table 3:** Data of clinical course

	GHB-patients N = 39
Delirium in %	30.8
Seizure in %	0
Referral to intensive care unit in %	20.5
Mechanical ventilation in %	5.1
Premature treatment drop-out in %	46.2

All data was captured and evaluated by an experienced physician. Statistical analyses were carried out using SAS (statistical analysis system) software by SAS Institute. For bivariate comparisons Fisher’s exact test or a t-test were used.

## Results

### Sample characteristics

43 GHB-patients were screened for study participation. Two did not consent and so we could include 41 patients. Two patients withdrew their consent during their hospital stay leaving 39 patients for analysis.

#### Socioeconomic characteristics of GHB-patients

Data are given in Table 1. GHB-patients were equally distributed for sex. GHB-patients on average had good school education. 46.2% had 13 years of schooling (this is the longest possible regular school attendance in Germany). Regarding post-school education, 59% of the sample had successfully finished either an apprenticeship or a university degree. One third of the sample had regular employment.

#### Data on clinical history

The mean GHB consumption (data not shown in the tables) within 24 h was 31.61 ml (standard deviation 18.00 ml, minimum 4 ml, maximum 80 ml). All other data are given in Table 2. One third (33.3%) of the GHB-patients fulfilled the criteria for additional active substance use disorder. Six of the GHB patients (15.7%) had an HIV infection (data not shown in the tables). All of these cases were male. All six reported additional amphetamine consumption and three of them fulfilled the criteria for amphetamine addiction. One case had a third active addictive disease. Only 5% of the patients had a history of withdrawal seizures during previous detoxifications. In contrast the GHB-patients had a high rate of history of delirium during previous detoxifications.

#### Clinical course of GHB-patients

GHB withdrawal was treated using diazepam reduction regimes. The mean peak of diazepam use within 24 h was 76.66 mg (standard deviation 23.09 (data not shown in the tables)). Table 3 shows data on clinical course and complications. Almost a third (30.8%) of GHB-patients experienced delirium during detoxification. Every fifth (20.5%) patient of our study group needed intensive care treatment and 5.1% needed mechanical ventilation whereas no GHB-patient suffered a withdrawal seizure. Premature drop out among GHB-patients was 46.2%.

The mean daily intake of GHB was statistically significantly (p = 0.0225) higher in patients with a delirium during detoxification (mean 41.3, SD 19.4) than in patients who did not suffer a delirium during detoxification (mean 27.3, SD 15.9). In a logistic regression analysis with occurrence of a withdrawal delirium as the dependent and daily GHB dosage as the independent variable, GHB dose in this small sample failed to be a statistically significant risk factor for the occurrence of a delirium (likelihood-ratio χ2-test: p = 0.0633).

## Discussion

### Socioeconomic status of GHB-patients

In the current clinical sample, GHB seems to be a drug used by people of both sexes in their late twenties/early thirties. These users seem to be well educated as demonstrated by the high percentage who finished 13 years of school. Almost 60% of the patients had finished an apprenticeship or gained a university degree. However more than 50% of the patients had no regular income (either supported by parents, or receiving welfare, or unemployed). This might indicate that use of GHB makes it more difficult to convert good educational achievements into the expected occupational status. The GHB users were less likely to live with a partner or to have a child. But there might be reasons other than drug use which explain these findings. The socioeconomic status of our GHB-patients is in parts similar to that reported by Raposo Pereira et al. [12] In their sample of N = 81 participants they found the average GHB user to be young, mainly single, living alone, well-educated, and generally studying. This might help to build literature on persons using GHB. Raposo Pereira and colleagues intentionally included male patients only, claiming that most GHB-users are male. We could not confirm this as in our sample sex was almost equally distributed. In a multicentre observational study, Wolf and colleagues [21] had found 27% of their sample to be female.

### Clinical history

Six of our GHB-patients (15.7%) had an HIV infection. All of these were male and reported additional amphetamine consumption. This could indicate the use of GHB in situations described in the literature as “chemsex”. This means the combining of sex and drugs, in particular the synthetic amphetamine mephedrone, GHB, ketamine and crystal methamphetamine within extended sexual sessions involving multiple partners. The risk of getting infected with a sexually transferred disease is high [22]. Hammoud and colleagues [23] conducted an online prospective observational study of Australian gay and bisexual men. Being HIV-positive, having more gay friends who use drugs, a greater number of sexual partners, group sex, and unsafe sex with casual partners was independently associated with GHB use in the last 6 months. The fact that this pattern held true for only about a sixth of our subjects suggests that chemsex participants are only a small subgroup of GHB-users.

### Clinical course and complications

The clinical course of detoxification treatment of our GHB-patients showed a high rate of complications withdrawing from the drug. Most significantly almost a third developed delirium (30.8%). Patients had a high risk of needing intensive care treatment (20.5%) including artificial ventilation (5.1%). McDonough et al. [24] conducted a literature review of 38 cases who suffered GHB withdrawal syndrome. Delirium was reported in 53% of the cases. No withdrawal seizures were recorded. This is matching our findings. In an observational study from Belgium [25] (N = 42) GHB-addicted patients were treated with benzodiazepines at tapered dosage. Delirium occurred in 21% (N = 9) of patients. Two of them (4.7%) needed intensive care treatment. The rate of delirium in this study is lower than our findings, and the rate of transfer to intensive care is even lower. There is no information available on the decision-making process around the transfer of patients to ICU. This might reflect different standards of decision-making in different hospitals or countries. We had no standards for decision-making to decide who was in need of intensive care. The decision for transfer to ICU was made by the responsible doctor based on clinical presentation and might differ from other clinical settings. 13.2% of our patients reported ICU treatment during withdrawal in a variety of hospitals across Germany, before being admitted to our hospital. These differences between countries in rates of ICU admissions might reflect different protocols and should be investigated further. Nevertheless, the high rates of ICU treatment serve as an indicator of the complications of GHB withdrawal.

### Model of delirium

It would be of high interest to identify more details of the pathophysiological pathways leading to delirium in GHB-withdrawal. The Neurotransmitter-Hypothesis of delirium suggests that the most commonly described neurotransmitter changes associated with delirium are reduced availability of acetylcholine, excess release of dopamine, norepinephrine, and/or glutamate, and alterations (e.g., both a decreased activity and an increased activity depending on circumstances and etiological factors) in serotonin, histamine, and/or gamma‐aminobutyric acid [14].

As of to date we have no specific model of mechanism for delirium during GHB withdrawal. Due to its strong sedative effect, the mechanism might be similar to alcohol and benzodiazepines. Withdrawal of alcohol (and benzodiazepines) has been related to transiently reduced GABA inhibitory function [26]. The GABA receptor is a postsynaptic receptor complex with specific receptors for GABA, benzodiazepines, barbiturates, and perhaps alcohol [26]. Benzodiazepines, and possibly alcohol, enhance GABA inhibitory activity by increasing GABA receptor binding. Withdrawal of alcohol, which may result in hyperalert-hyperactive delirium, may be associated with reduced GABAergic activity [26]. Thus, alcohol withdrawal delirium may be the result of imbalanced neurotransmission, caused by over activity of the noradrenergic system, decreased function of the serotonergic system, and reduced GABA inhibitory function [27]. GHB-induced delirium might be following similar pathways. However, this matter has been a subject of debate [28]. Nevertheless, similar to alcohol or benzodiazepines the occurrence of delirium states may be associated with a sudden reduced GABAergic activity during GHB withdrawal. However, the question remains yet to be answered why the risk of delirium seems to be so much higher than during alcohol withdrawal. A t-test revealed that subjects with delirium in the clinical course had a statistically significant higher intake of GHB as compared to patients without delirium (p = 0.0225). However, this finding has to be interpreted with caution, since logistic regression analysis with amount of intake of GHB and benzodiazepines as factors did not show a significant effect of these factors. Further research into this topic is needed to verify this finding.

### Dropouts

Given the dangerous course of GHB detoxification, the rate of premature treatment dropout was astonishing high. Substantial effort has been made in the literature to shed light on the reasons for non-compliance towards drug treatment programmes [29, 30], but none specifically regarding GHB users. In an earlier study, not including GHB users, [31], we had found the best positive predictors were being young, female, living with a partner, having children, being employed, and having a good educational status. All of these indicating a supportive social network, which helps to finish our detoxification treatment. Having children increased the probability of successful QDT, maybe due motivation as a result of responsibility for the children’s wellbeing. Family ties might be a significant motivational predictor for treatment outcome.

### Strengths and limitations

Our patients were evaluated in one single institution, with the same treatment routines and a high number of patients. This is a strength. The study was interrupted due to the coronavirus pandemic. This is a weakness. The data was collected in one single hospital which is a strength and a weakness at the same time. A strength is the homogenous treatment. A weakness is that our findings cannot be generalised. Our findings can help to generate hypotheses for further studies though.

## Conclusions

Detoxification of GHB seems to be complicated and dangerous. The mean dosage of benzodiazepines needed to control withdrawal symptoms is high. The prevalence of a delirium and need for transferal to intensive care unit during detoxification treatment of GHB is extraordinarily high, even under appropriate clinical treatment, the reasons remaining unknown. Therefore an intensive care unit should be available when GHB detoxification is carried out. Further studies need to evaluate the possibilities of prophylactic treatment of delirium during detoxification.

## Data Availability

The datasets used and/or analysed during the current study are available from the corresponding author on reasonable request.
